# Supplementary material for: Into the Deep (Sequence) of the Foot-and-Mouth Disease Virus Gene Pool: Bottlenecks and Adaptation during Infection in Naïve and Vaccinated Cattle
Source: Pathogens. 2020 Mar 12;9(3):208. doi: 10.3390/pathogens9030208 (PMC7157448; doi:10.3390/pathogens9030208)
Supplement: Supplementary file 1 [file pathogens-09-00208-s001.zip › FigS1.pdf]

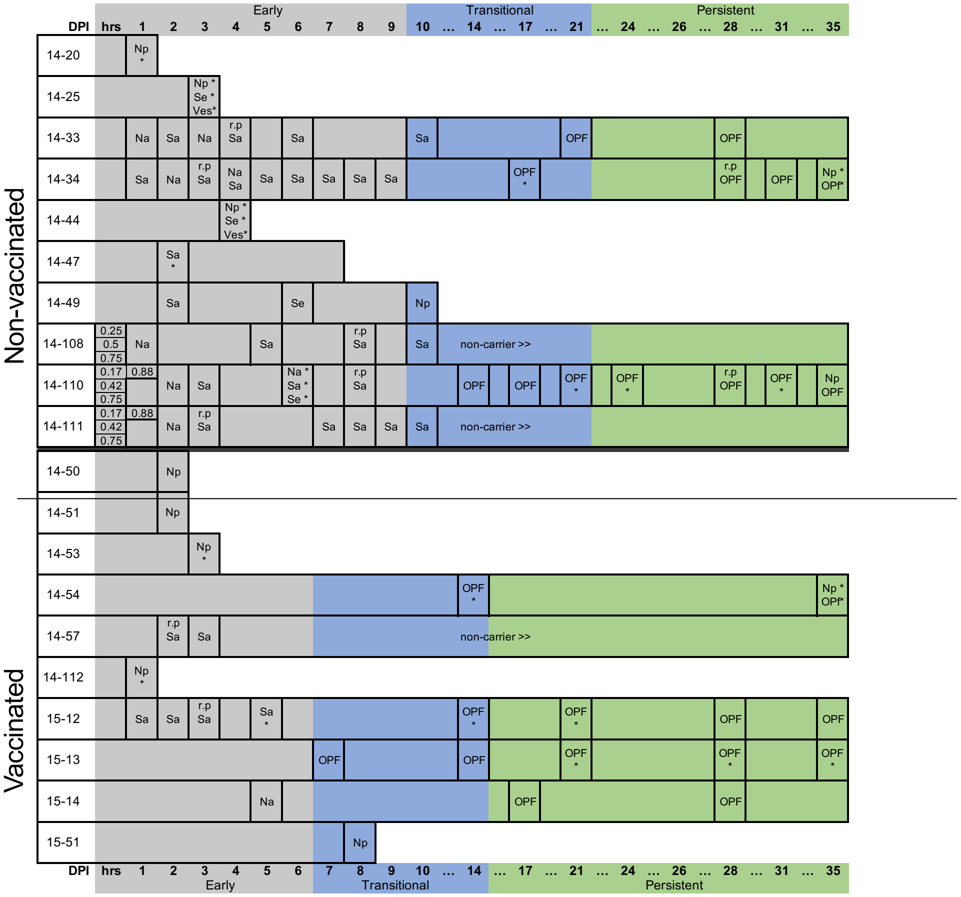

animal	dpi	sample type	GCN-sample	GCN-serum
14-108	0.25	nasal	4.69	na†
14-108	0.5	nasal	4.39	na
14-108	0.75	nasal	4.64	na
14-108	1	nasal	5.64	Neg#
14-108	3	nasal	7.65	3.37
14-108	5	saliva	11.21	6.59
14-108	8	saliva	8.11	Neg
14-108	10	saliva	7.32	Neg
14-110	0.17	nasal	6.01	na
14-110	0.42	nasal	4.50	na
14-110	0.75	nasal	4.72	na
14-110	0.88	nasal	4.94	na
14-110	2	nasal	5.41	Neg
14-110	3	saliva	5.88	Neg
14-110	6	nasal	8.70	6.64
14-110	6	saliva	10.49	6.64
14-110	6	serum	6.64	6.64
14-110	8	saliva	8.71	Neg
14-110	14	OPF	4.62	Neg
14-110	17	OPF	5.23	Neg
14-110	21	OPF	5.26	Neg
14-110	24	OPF	5.09	Neg
14-110	28	OPF	5.24	Neg
14-110	31	OPF	4.20	Neg
14-110	35	Np	4.68	Neg
14-110	35	OPF	5.20	Neg
14-111	0.17	nasal	6.40	na
14-111	0.42	nasal	4.44	na
14-111	0.75	nasal	5.49	na
14-111	0.88	nasal	7.06	na
14-111	2	nasal	5.67	Neg
14-111	3	saliva	6.70	Neg
14-111	7	saliva	8.51	6.28
14-111	8	saliva	6.99	3.25
14-111	9	saliva	6.49	Neg
14-111	10	saliva	6.13	Neg
14-112	1	Np	3.57	Neg
14-20	1	Np	5.00	Neg
14-25	3	Np	4.61	7.94
14-25	3	serum	7.94	7.94
14-25	3	vesicle	6.54	7.94
14-33	1	nasal	4.35	Neg
14-33	2	saliva	4.80	Neg
14-33	3	nasal	5.26	Neg
14-33	4	saliva	6.65	4.71
14-33	6	saliva	9.36	4.05
14-33	10	saliva	8.37	Neg
14-33	21	OPF	4.19	Neg
14-33	28	OPF	4.78	na

animal	dpi	sample type	GCN-sample	GCN-serum
14-34	1	saliva	3.81	Neg
14-34	2	nasal	4.41	Neg
14-34	3	saliva	6.69	6.99
14-34	4	nasal	9.35	8.51
14-34	4	saliva	8.13	8.51
14-34	5	saliva	8.87	7.49
14-34	6	saliva	8.15	3.63
14-34	7	saliva	7.20	3.49
14-34	8	saliva	6.21	Neg
14-34	9	saliva	6.82	Neg
14-34	17	OPF	5.23	na
14-34	28	OPF	4.73	Neg
14-34	31	OPF	4.82	Neg
14-34	35	Np	5.31	Neg
14-34	35	OPF	5.31	Neg
14-44	4	Np	4.20	8.37
14-44	4	serum	8.37	8.37
14-44	4	vesicle	8.89	8.37
14-47	2	saliva	5.91	Neg
14-49	2	saliva	6.10	Neg
14-49	6	serum	6.60	6.60
14-49	10	Np	4.05	Neg
14-50	2	Np	3.69	Neg
14-51	2	Np	4.80	Neg
14-53	3	Np	4.37	Neg
14-54	14	OPF	5.25	Neg
14-54	35	Np	5.20	Neg
14-54	35	OPF	4.54	Neg
14-57	2	saliva	4.13	Neg
14-57	3	saliva	4.60	Neg
15-12	1	saliva	5.28	Neg
15-12	2	saliva	4.03	Neg
15-12	3	saliva	5.32	Neg
15-12	5	saliva	5.06	Neg
15-12	14	OPF	4.56	Neg
15-12	21	OPF	4.88	Neg
15-12	28	OPF	4.71	Neg
15-12	35	OPF	3.53	Neg
15-13	7	OPF	5.61	Neg
15-13	14	OPF	5.03	Neg
15-13	21	OPF	4.52	Neg
15-13	28	OPF	6.16	Neg
15-13	35	OPF	4.87	Neg
15-14	5	nasal	4.27	Neg
15-14	17	OPF	3.42	Neg
15-14	28	OPF	4.82	Neg
15-51	8	Np	3.25	Neg

Figure S1. Experimental design: sequenced sample sources and times. Naïve cattle above center black line and vaccinated below. Abbreviations: hrs - fraction of first 24 hours (all nasal secretions), dpi – days post infection, Na – nasal secretion, Np – nasopharyngeal tissue (necropsy), OPF – oropharyngeal fluid, Sa – saliva, Se – serum, Ves – epithelial vesicle, r.p – both passaged and un-passaged samples were sequenced. * consensus sequence available only (no deep sequencing data). **Table**: Genome copy numbers (GCN) of samples and sera for each sample. GCN = log10 FMDV genome copies, /mg for tissue and /mL for liquids. na† - not measured. Neg# - below the level of detection (1.53 log_{10} for tissue, 1.57 log_{10} for liquid samples)
