# Supplementary material for: Into the Deep (Sequence) of the Foot-and-Mouth Disease Virus Gene Pool: Bottlenecks and Adaptation during Infection in Naïve and Vaccinated Cattle
Source: Pathogens. 2020 Mar 12;9(3):208. doi: 10.3390/pathogens9030208 (PMC7157448; doi:10.3390/pathogens9030208)
Supplement: Supplementary file 1 [file pathogens-09-00208-s001.zip › FigS2.pdf]

	Early Subs Rate - Naïve			Transitional Subs Rate - Naïve			Persistent Subs Rate - Naïve		
	All Subs	Nonsyn Subs	Syn Subs	All Subs	Nonsyn Subs	Syn Subs	All Subs	Nonsyn Subs	Syn Subs
subs/day	3.61[a]	1.52[b]	2.14	2.44	0.70	1.74	1.54[a]	0.28[b]	1.27
subs/site/year	0.188[a]	0.079[b]	0.111	0.127	0.037	0.091	0.080[a]	0.014[b]	0.066
	Early Subs Rate - Vaccinated			Transitional Subs Rate - Vaccinated			Persistent Subs Rate - Vaccinated		
subs/day	2.51	1.07	1.43	1.70	0.73	0.97	1.52	0.49	1.03
subs/site/year	0.131	0.056	0.075	0.089	0.038	0.050	0.079	0.026	0.054

	Trans-Persistent Subs Rate - Naïve			1-10 dpi Subs Rates - Terminators		
	All Subs	Nonsyn Subs	Syn Subs	All Subs	Nonsyn Subs	Syn Subs
subs/day	1.90	0.46	1.44	4.29	1.92	2.50
subs/site/year	0.099	0.024	0.075	0.224	0.100	0.131
	Trans-Persistent Subs Rate - Vaccinated			1-10 dpi Subs Rates - Carriers		
subs/day	1.53	0.54	0.99	3.47	1.45	2.02
subs/site/year	0.080	0.028	0.051	0.181	0.076	0.105

Figure S2. Pairwise differences: Vaccinated vs. Naïve, Carriers vs. Terminators. The number of consensus-level pairwise differences between each sample and the preceding sample (inoculum = 0 dpi) divided by intervening time within each animal. Pairwise values from 0.25 - 0.88 dpi and animals of unknown carrier status omitted. For instances of samples with same dpi - same animal (different tissue) and where both raw and passaged were sequenced, pairwise differences were averaged. [a] = $P < 0.05$ and [b] = $P < 0.005$.
