# Supplementary material for: Into the Deep (Sequence) of the Foot-and-Mouth Disease Virus Gene Pool: Bottlenecks and Adaptation during Infection in Naïve and Vaccinated Cattle
Source: Pathogens. 2020 Mar 12;9(3):208. doi: 10.3390/pathogens9030208 (PMC7157448; doi:10.3390/pathogens9030208)
Supplement: Supplementary file 1 [file pathogens-09-00208-s001.zip › FigS3.pdf]

Figure S3. Amino acid alignment of all sample consensus sequences. Inoculum used as reference.

	Gene enumeration	4	8	11	19	32	34	81	123	146	188	44	78	82	88	101	131	181	191	195	70	75	99	131	139	175	179	190	208	220	28	32	33	96	142	144	147	155	160	196	197	199	3	45	85	43	73	84	133	136	224	248	279	280	283	288	301	3	17	123	133	136	142	147	4	19	34	40	66	91	169	3	61	138	150	177	378	463
	Nucleotide enumeration	10	22	32	55	95	100	241	369	436	562	988	1090	1102	1120	1160	1250	1400	1429	1441	1720/1	1736	1807	1903/4	1928	2036	2048	2080	2135	2171	2258/9	2269	2272	2461	2599	2605/7	2615	2638/9	2654	2762	2765	2771	2816	2996	3115	3452	3541	3574	3722	3732	3995	4067	4160	4163	4171	4187	4225	4285	4628	4645	4676	4684	4703	4718	4748	4792	4838	4856	5148	5221	5455	5596	5771	6002	6039	6119	6721	6976
	CDS enumeration	4	8	11	19	32	34	81	123	146	188	330	364	368	374	387	417	467	477	481	574	579	603	635	643	679	683	694	712	724	753	757	758	821	867	869	872	880	885	921	922	924	939	999	1039	1151	1181	1192	1241	1244	1332	1356	1387	1388	1391	1396	1409	1429	1543	1549	1559	1562	1568	1573	1583	1598	1613	1619	1716	1741	1819	1866	1924	2001	2013	2040	2241	2326
	Gene region	Lpro										VP2									VP3										VP1												2A	2B		2C												3A							3B				3C			3D						
	Inoculum	T	I	V	A	T	H	E	E	Q	L	A	L	E	H	M	E	V	N	A	D	A	T	E	R	T	I	I	K	Q	H	I	G	S	G	S	T	V	A	S	S	D	N	T	L	T	A	H	G	D	N	I	M	Q	V	P	I	I	S	G	K	N	P	E	A	L	K	A	D	H	A	I	K	T	M	K	H	V
Non-vaccinated	14-20_1_dpi_Np																							K																															M				N																			
	14-25_3_dpi_Np																																				M																																									?
	14-25_3_dpi_Ves																																				M																										D																?
	14-25_3_dpi_Se																																				M																																									?
	14-33_1_dpi_Na																																				M																										D																
	14-33_2_dpi_Sa																																				M																																									
	14-33_3_dpi_Na																							K																															M				N																			
	14-33_4_dpi_Sa																							K																															M				N																			
	14-33_4_dpi_Sa_raw																																				M																										D																
	14-33_6_dpi_Sa																																				M																										D																
	14-33_10_dpi_Sa_raw																																				M																										D					R												
	14-33_21_dpi_OPF																																S			R	M																										D																
	14-33_28_dpi_OPF																																S			R	M																				L						D																
	14-34_1_dpi_Sa																																					A																		M																								
	14-34_2_dpi_Na																																					A																		M																								
	14-34_3_dpi_Sa																							K																															M				N																			
	14-34_3_dpi_Sa_raw																																				M																										D																
	14-34_4_dpi_Na																																				M																										D																
	14-34_4_dpi_Sa																																				M																										D																?
	14-34_5_dpi_Sa																																				M																							N																			
	14-34_6_dpi_Sa																			A																	M																										D																
	14-34_7_dpi_Sa																																			R										M		N		E		T			M																								
	14-34_8_dpi_Sa																																	T		R				F						M	T	N		E		T			M																								
	14-34_9_dpi_Sa																																			R										M		N		E		T			M																								
	14-34_17_dpi_OPF																																			R																			M																								
	14-34_28_dpi_OPF																																			R																			M																								
	14-34_28_dpi_OPF_raw																																			R																			M																								
	14-34_31_dpi_OPF			A																																R		V																	M							R																	
	14-34_35_dpi_Np			A																					K			V								R		V																	M							R																?
	14-34_35_dpi_OPF																																			R																			M																							?
	14-44_4_dpi_CBV																							K																															M				N																			
	14-44_4_dpi_Np																							K																															M				N																			
	14-44_4_dpi_Se																																																						M																							?
	14-47_2_dpi_Sa																																				M																										D																?
	14-49_2_dpi_Sa																																				M																																									
	14-49_6_dpi_Se																																				M																																									
	14-49_10_dpi_Np																		T												Q					R					L	G				M		N		E		T			M					S																			
	14-108_0.25_dpi_Na																							K																															M				N																			
	14-108_0.5_dpi_Na																							K																															M				N																			
	14-108_0.75_dpi_Na																		T																		M																										D																
	14-108_1_dpi_Na																																				M																																									
	14-108_3_dpi_Na																																				M																										D																
	14-108_5_dpi_Sa																																				M																										D																
	14-108_8_dpi_Sa_raw																																				M																										D																
	14-108_8_dpi_Sa																																				M																										D																
	14-108_10_dpi_Sa																													R						R	M																										D			V													
	14-110_0.17_dpi_Na																	A																				A							V										M																								
	14-110_0.42_dpi_Na																							K																															M				N																			
	14-110_0.75_dpi_Na																							K																															M				N																			
	14-110_0.88_dpi_Na																																																						M																								
	14-110_2_dpi_Na																																				M																																									
	14-110_3_dpi_Sa							K																													M																										D																
	14-110_6_dpi_Na																														R						M							I																																			
	14-110_6_dpi_Sa																														R						M							I																																			
	14-110_6_dpi_Se																														R						M							I																																		?
	14-110_8_dpi_Sa																														R						M							I																																			
	14-110_14_dpi_OPF											T												K												R	M																		M				N																			
	14-110_17_dpi_OPF											T												K												R	M																		M							D												I			
	14-110_21_dpi_OPF											T									N		A													R	M																									D																?
	14-110_24_dpi_OPF											T									N		A													R	M																									D																?
	14-110_28_dpi_OPF											T									N		A													R	M																									D																
	14-110_28_dpi_OPF_raw											T									N		A													R	M																									D																
	14-110_31_dpi_OPF											T									N		A													R	M																									D		A														?
	14-110_35_dpi_Np											T									N		A													R	M																									D																?
	14-110_35_dpi_OPF											T									N		A						R							R	M																									D		A														
	14-111_0.17_dpi_Na																							K																															M				N																			
	14-111_0.42_dpi_Na																																				M																										D																
	14-111_0.75_dpi_Na																							K																															M																								
	14-111_0.88_dpi_Na																																				M																										D																
	14-111_2_dpi_Na																																		R		M																																					R					
	14-111_3_dpi_Sa																																				M																										D																
	14-111_3_dpi_Sa_raw																																				M																										D																
	14-111_7_dpi_Sa																																		R		M																																					R					
	14-111_8_dpi_Sa																																		R		M																																					R					
	14-111_9_dpi_Sa																																		R		M																														F							R					
	14-111_10_dpi_Sa		V																											R							M																										D																
Vaccinated	14-50_2_dpi_Np																																				M																										D																
	14-51_2_dpi_Np																																				M																										D																
	14-53_3_dpi_Np																																				M																																									?
	14-54_14_dpi_OPF																																			R	M																									D																?
	14-54_35_dpi_Np						Y																				T									R	M																									D	L															?
	14-54_35_dpi_OPF						Y																													R	M									M																	D																?
	14-57_2_dpi_Sa										F																										M																	R									D										T							
	14-57_3_dpi_Sa												M																								M																										D										T							
	14-112_1_dpi_Np																																				M																																									
	15-12_1_dpi_Sa																																				M																										D							E									
	15-12_2_dpi_Sa																																				M																										D							E									
	15-12_3_dpi_Sa																																				M																										D							E									
	15-12_3_dpi_Sa_raw																																				M																										D							E									
	15-12_5_dpi_Sa																									K											M																										D																
	15-12_14_dpi_OPF													K																							M																										D							E									?
	15-12_21_dpi_OPF	A								E				K																							M																				V					D							E	Y								?
	15-12_28_dpi_OPF														N																						M	A																								D							E			V		I				
	15-12_35_dpi_OPF				T										N																						M	A													S												D							E			V		I			Y	
	15-13_7_dpi_OPF																							K													M						S												M				N																			
	15-13_14_dpi_OPF																							G													M					G																				D												I			
	15-13_21_dpi_OPF								D															K													M						S												M			V	N																		?
	15-13_28_dpi_OPF																							K													M						S												M				N							?	?	?										?
	15-13_35_dpi_OPF																G							G													M																									D														?	?
	15-14_5_dpi_Na															T										K											M																										D																
	15-14_17_dpi_OPF					I																V										V				R	M																																V										M
	15-14_28_dpi_OPF					I																														R	M																																V										
	15-51_8_dpi_Np																		D																		M	M											D				T										D												I		R		
